# The landscape of tiered regulation of breast cancer cell metabolism

**DOI:** 10.1038/s41598-019-54221-y

**Published:** 2019-11-28

**Authors:** Rotem Katzir, Ibrahim H. Polat, Michal Harel, Shir Katz, Carles Foguet, Vitaly A. Selivanov, Philippe Sabatier, Marta Cascante, Tamar Geiger, Eytan Ruppin

**Affiliations:** 1 Center for BioInformatics and Computational Biology, Dept. of Computer Science and the University of Maryland Institute of Advanced Computer Studies (UMIACS), University of Maryland, College Park, MD 20742 USA; 2 Department of Biochemistry and Molecular Biomedicine, Faculty of Biology, Universitat de Barcelona and Institut de Biomedicina de la Universitat de Barcelona (IBUB), Barcelona, Spain; 3 Centro de Investigación Biomédica en Red de Enfermedades Hepáticas y Digestivas (CIBEREHD), Instituto de Salud Carlos III (ISCIII), Madrid, Spain; 4 Equipe environnement et prédiction de la santé des populations, Laboratoire TIMC (UMR 5525), CHU de Grenoble, Université Grenoble Alpes, La Tronche, France; 5 Department of Human Molecular Genetics and Biochemistry, Sackler Faculty of medicine, Tel Aviv University, Tel Aviv, Israel; 6 Cancer Data Science Lab, National Cancer Institute, NIH, Bethesda, MD USA

**Keywords:** Cancer genomics, Computational models, Regulatory networks

## Abstract

Altered metabolism is a hallmark of cancer, but little is still known about its regulation. In this study, we measure transcriptomic, proteomic, phospho-proteomic and fluxomics data in a breast cancer cell-line (MCF7) across three different growth conditions. Integrating these multiomics data within a genome scale human metabolic model in combination with machine learning, we systematically chart the different layers of metabolic regulation in breast cancer cells, predicting which enzymes and pathways are regulated at which level. We distinguish between two types of reactions, directly and indirectly regulated. *Directly-regulated* reactions include those whose flux is regulated by transcriptomic alterations (~890) or via proteomic or phospho-proteomics alterations (~140) in the enzymes catalyzing them. We term the reactions that currently lack evidence for direct regulation as (putative) *indirectly regulated* (~930). Many metabolic pathways are predicted to be regulated at different levels, and those may change at different media conditions. Remarkably, we find that the flux of predicted indirectly regulated reactions is strongly coupled to the flux of the predicted directly regulated ones, uncovering a tiered hierarchical organization of breast cancer cell metabolism. Furthermore, the predicted indirectly regulated reactions are predominantly reversible. Taken together, this architecture may facilitate rapid and efficient metabolic reprogramming in response to the varying environmental conditions incurred by the tumor cells. The approach presented lays a conceptual and computational basis for mapping metabolic regulation in additional cancers.

## Introduction

Cancer cells adapt their metabolism to facilitate biomass formation to support their rapid proliferation. Transcriptional regulation alone does not account for many of the metabolic alterations observed in cancer^1,2^, suggesting that post-transcriptional, post-translational and protein phosphorylation mechanisms may play an important role in modulating cancer metabolism and determining cancer cell phenotypes^3–6^. Here we aim to chart the transcriptional, post-transcriptional and post-translational regulation of MCF7 breast cancer cell metabolism on a genome scale. This is performed via measurements of multi-omics data employing MCF7 breast cancer cells under three different *in vitro* growth conditions, and its analysis via an integration of this data within a genome scale metabolic model (GSMM) of human metabolism. Our approach is inspired by previous large-scale omics studies of the multi-level regulation of bacterial metabolism^7–9^ and yeast^10^, which have advanced our understanding of the organization and regulation of metabolism in these organisms.

Genome scale metabolic modeling is an increasingly widely used computational framework for studying metabolism. Given the GSMM of a species alongside contextual information such as growth media and omics data, it has been shown that one can fairly reliably predict numerous metabolic phenotypes, including cells’ growth rates, metabolite uptake and secretion rates and internal fluxes, gene essentiality, and more. Over the last few years, GSMMs have successfully served as a basis for many computational studies of cancer, e.g.^11–16^. GSMMs have also been used to predict post-transcriptional regulation of metabolic enzymes in healthy tissues^17^ but going beyond that to systematically analyze metabolic regulation in cancer is addressed here for the first time to the best of our knowledge.

## Results

### Data collection and preliminary model-free analysis

We collected omics measurements in MCF7, a breast cancer cell line, grown under three different conditions: (1) Minimum Essential Medium (MEM) with glucose and without glutamine (MEM-Gln), (2) MEM with glucose and glutamine (MEM) and (3) MEM with glucose, glutamine and supplemented with Oligomycin – an inhibitor of ATP synthase that inhibits cell respiration (MEM+Oli). The media were chosen because they reflect multiple stress conditions for the cell: one media (glutamine deprivation) is chosen because MCF7 cells rely on glutamine as the main source of energy, and the other media (supplement of Oligomycin) is chosen because it emulates tumor hypoxic conditions.

**Figure 1:**
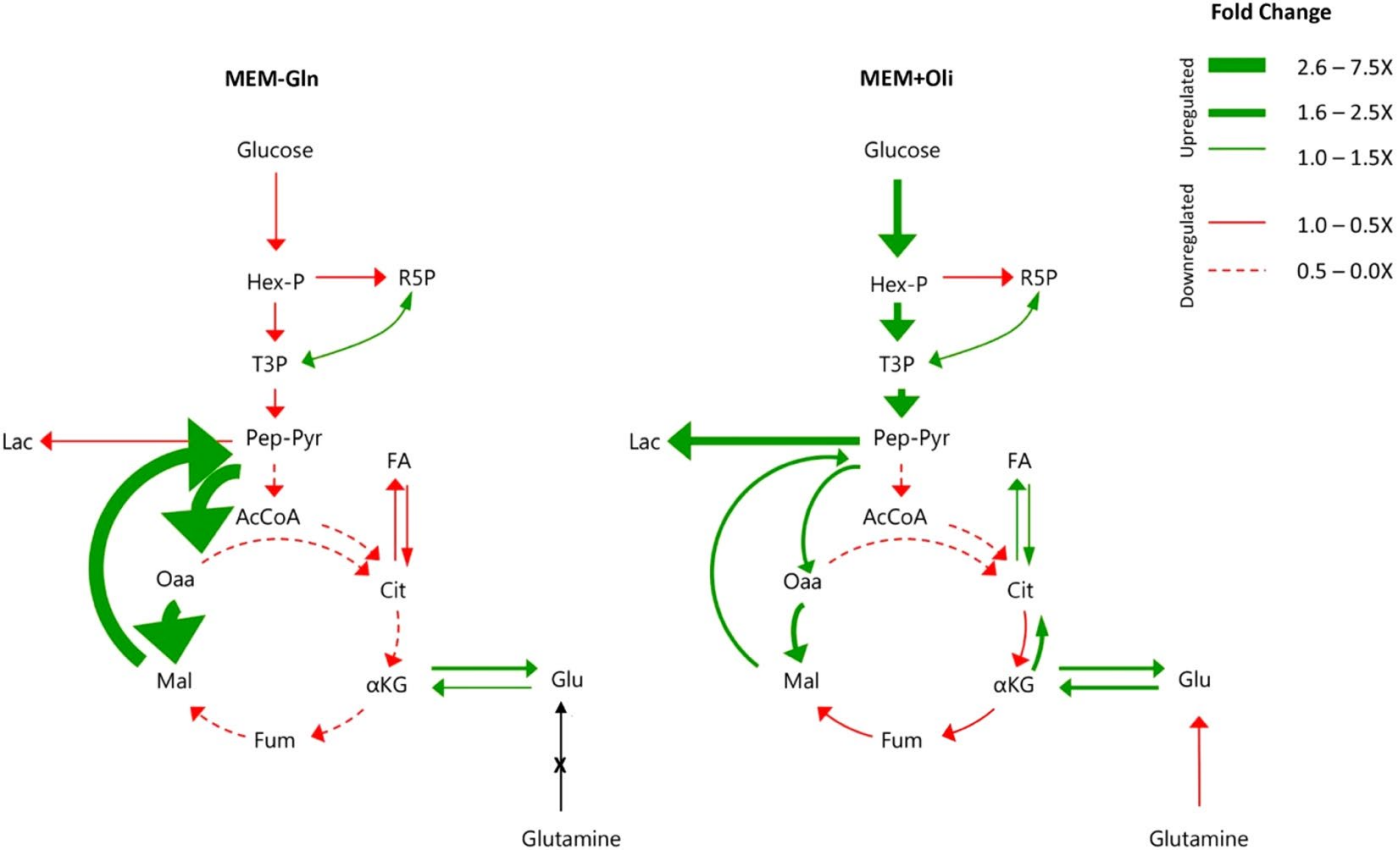
Metabolic flux map of MCF7 breast cancer cells under MEM-Gln or MEM+Oli growth conditions compared to MEM condition. The fluxes were estimated by using Isodyn software. In each growth condition, the calculated flux was normalized against the flux of MEM growth condition in order to calculate the net change.

The measurements were repeated twice under each condition at two time points - after 8 and 24 hours, resulting in overall 6 × 2 multi-omics datasets. Each such dataset includes the gene-expression of 1372 metabolic genes, proteomics for 486 metabolic enzymes (~97% of the measured enzymes have gene expression values), phosphorylation values for 71 phosphorylation sites on metabolic enzymes, and flux measurements of 44 metabolic reactions (see methods). To obtain flux measurements, we fitted all the data obtained through spectrophotometric measurements and ^13^C assisted metabolomics experiments using our in-house developed software that simulates dynamics of metabolites ^13^C labeling, Isodyn^18–22^. Fitting the data allows determining the metabolic flux profiles of MCF7 breast cancer cells under three different growth conditions (see methods). Figure 1 summarizes the qualitative changes in the metabolites and their analysis using Isodyn. The analysis demonstrates a decrease in the fluxes of glycolysis, lactate production, pentose phosphate pathway (PPP) activity, tricarboxylic acid cycle (TCA) cycle utilization and fatty acid synthesis when the cells are at MEM-Gln growth condition compared to MEM. Moreover, increased pyruvate cycle, which is the conversion of pyruvate to oxaloacetate via pyruvate carboxylase followed by its conversion to malate and consequently back to pyruvate via malic enzyme, occurs mainly in MCF7 cells at MEM-Gln condition compared to the MEM growth condition. On the other hand, in the MEM+Oli growth condition, increased glycolysis, lactic acid fermentation and pyruvate cycle are observed compared to the MEM growth condition, together with decreased TCA cycle activity, PPP and lipogenesis. All measured and estimated fluxes and their values are listed in SI Table 1.

To obtain a genome wide view of pathway-level differences in the transcriptional data across the different growth conditions, we first compared (using a t-test) the metabolic gene expression values between the different growth conditions to identify metabolic pathways that were significantly up or down regulated in any of these conditions compared to the others. We found that upon oligomycin treatment, carnitine shuttle pathway is downregulated compared to the other growth conditions, as well as the urea cycle/amino group metabolism pathway. On the other hand, fatty acid activation and C5-Branched dibasic acid metabolism (among other pathways) were found to be elevated upon such treatment - a full table listing the significant growth condition-specific changes is provided in SI Table 2(a–c), all p-values were FDR corrected for 0.05). A similar analysis of the proteomics data revealed different results. While carnitine shuttle pathway activation was consistent with the gene expression analysis, the fatty acid pathways (activation, elongation and oxidation) were now found to be downregulated upon Oligomycin treatment. These results, consistent with previous observations both in yeast^23,24^ and in human^25,26^, point to the significant differences between the mRNA and protein levels of many metabolic enzymes and call for a systematic study of their potential functional regulatory implications.

### Overview of the metabolic modeling based analysis

**Figure 2:**
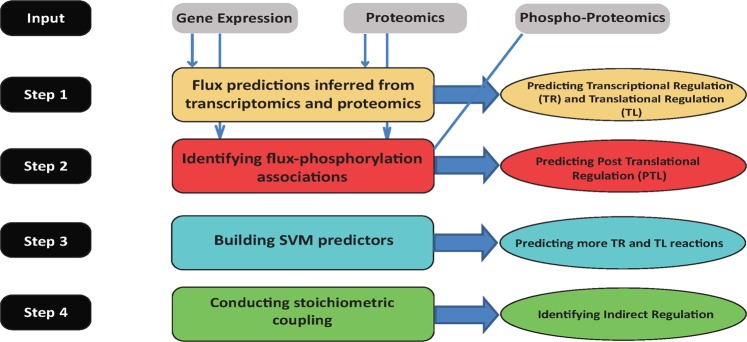
Systematic identification of reactions’ regulation: Step 1: Using gene-expression and proteomics data to predict transcriptionally and translationally regulated reactions. Step 2: Using phospho-proteomic data to predict post-translationally regulated reactions. Step 3: Based on the results of step 1, build predictors of TR and TL regulation. Step 4: Identifying indirectly regulated reactions that are metabolically regulated via stoichiometric coupling.

Our main goal in this study is to use the measured multi-omics data to systematically chart the different layers of metabolic regulation in breast cancer cells that orchestrate the actual metabolic flux across the network’s reactions occurring in each growth condition. Ideally, measuring the actual fluxes in each condition directly via tracing experiments would be adequate, but obviously, this can currently be done only for a small number of fluxes that are mainly involving central cell metabolism. Hence, alternatively, we integrated the various omics data measured in each growth condition within a genome scale model of human metabolism^27^ to infer the likely metabolic fluxes given these data in a genome wide manner. After an initial validation of these predictions, we proceeded to compare the flux predictions of the resulting reactions to the corresponding enzymes’ omics data to identify their regulation. This is performed in a stepwise manner as follows (Fig. 2):

1. GSMM based identification of transcriptional and translational directly regulated reactions: We first identify reactions that are *directly regulated –* that is, reactions whose model-based predicted flux alterations across the different conditions studied can be accounted for by molecular alterations at any one of the levels measured: those include reactions that are primarily *transcriptionally regulated* and primarily *translationally regulated*. These assignments are done in a mutually exclusive manner, as follows: (1) *transcriptionally regulated reactions (TR)* are those reactions whose enzymes’ gene expression levels match the predicted fluxes. (2) *translationally regulated reactions (TL)* are those reactions whose predicted flux levels do not match their gene expression levels, but they match the protein levels of their enzymes.
2. GSMM based identification of post-translationally directly regulated reactions: *Post-translationally regulated reactions’ (PTL)* assignments are given to the reactions where both the enzymes’ gene expression and proteomics levels do not match the predicted flux levels but the predicted flux levels across the different growth condition can be significantly associated with changes in the phosphorylation levels of the enzymes.
3. Building machine learning predictors of additional directly regulated reactions: For the majority of the metabolic reactions, however, we did not find omics evidence testifying that they are directly regulated at any of these three levels. One major reason for that may be the limited scope of the proteomics and phospho-proteomics measurements. We, therefore, built machine learning based predictors of TR and TL regulation based on the reactions that have already been labeled as such via the model-based analysis in step (1). Then, we applied these predictors in a genome wide manner to further identify sets of reactions that are predicted to be TR or TL regulated (detailed below). We then performed various genome wide analyses to further test and validate the veracity of these predictions.
4. Identifying stochiometrically coupled, indirectly regulated reactions: Finally, even after this prediction step, a large set of reactions still remains unassigned and are labeled as *indirectly regulated*. A major likely source of such indirect regulation is *metabolic regulation*^28^, which manifests itself in the stoichiometric coupling of the fluxes of different reactions across the metabolic network, and which we study further using the human metabolic model.

Below we provide a detailed description of each of these four steps and the results they uncover.

### Step 1: Identifying transcriptionally regulated (TR) and translationally regulated (TL) reactions

We first aimed to predict the fluxes of the reactions in each condition, to determine which reactions are directly regulated and at what level they are regulated. To this end, we used iMAT (the integrative Metabolic Analysis Tool)^17^, a computational method that systematically predicts metabolic fluxes in a GSMM by incorporating omics data (transcriptomics and/or proteomics) that represent the activity level of the metabolic enzymes. iMAT considers the gene expression or protein levels as cues for the likelihood that the enzymes in question carry a metabolic flux in their associated reactions. It then leverages the GSMM to accumulate these cues into a global flux distribution that is stochiometrically consistent and maintains mass balance across the entire metabolic network (see methods).

**Figure 3:**
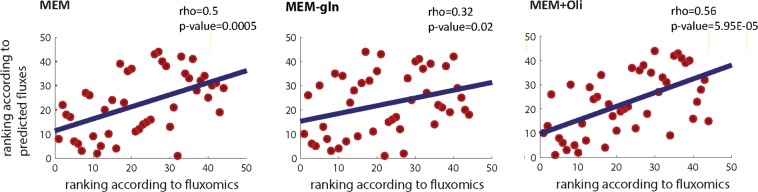
Scatter plot depicting the association between the measured and predicted fluxes in each of the three media conditions. Flux predictions were obtained by integrating the transcriptomics and proteomics data within the human metabolic model, as described in the main text.

To this end we first tested if the above described procedure yields flux predictions that are in accordance with those quantified with ^13^C Metabolic Flux Analysis (^13^C MFA). To this end, we combined both mRNA and protein expression measurements and used iMAT, a tool that extends upon the standard flux balance analysis (FBA) to predict the flux distribution that is the most likely given both types of data. Briefly, following a procedure already established and validated by^17^, the activity level of an enzyme was set according to the proteomics data when these data were available and according to the gene-expression otherwise, leaving the activity level unconstrained when large disparities existed between the gene expression and the proteomics data (see methods). Reassuringly, the accuracy of predicting the experimentally measured fluxes was significant across all growth conditions (Spearman correlation coefficient across all growth conditions = 0.42 p-values < 8.9671e-25, see Fig. 3 for the correlations obtained at each of the three different growth conditions).

Given these network wide flux predictions, we next set to identify the reactions that are *transcriptionally regulated (TR)*. To this end we discretized the gene expression measurements and the predicted fluxes into three levels of activity: low (TR-low), moderate (TR-moderate) and high (TR-high)). We then compared predicted flux level of each reaction to the discretized gene-expression level of the pertaining enzymes (see methods). Reactions whose predicted flux levels matched gene expression levels of their enzymes across the different measurements were considered to be TR. For the three conditions (MEM-Gln, MEM and MEM+Oli), 562, 550 and 556 reactions (approximately 28% of the model reactions) were identified as TR, respectively. Supporting these predictions, we found that the group of predicted TR reactions is enriched with transcription factor binding sites (using ENRICHR tool^29,30^, we calculated the enrichment according to several databases: Jaspar^31^ and Transfar^32^ (hyper-geometric p-value = 9.5892e-05), ChEA^33^ (hyper-geometric p-value = 1.2819e-10) and ENCODE^34,35^ (hyper-geometric p-value = 0.0029)) (see methods).

To predict translational regulation (TL), we searched for reactions whose (discretized) predicted flux activity levels were different from the transcriptomic levels of their enzymes. Such transcriptomic/flux ‘discordant’ reactions whose activity levels were high (low) according to the gene expression of their enzymes but low (high) according to the flux predictions are considered to be post-transcriptionally down-(up-)regulated. The correlation between the proteomics data and the predicted fluxes for this subset of TL predicted reactions was high and significant (rho = 0.75, 0.6, 0.5, for the 3 growth conditions, all p-values < 0.0071), as would be expected (SI Fig. 1). It is important to note that in order to avoid circularity, this correlation was calculated in a cross-validation manner only for sub-group which was not constrained in the algorithm input. Among the reactions identified as post-transcriptionally regulated, we denoted the subset of reactions whose predicted flux state highly matches the proteomics (discretized) levels in a given growth condition as *translationally (TL)-regulated*. Among those, about 15 reactions are predicted to be TL-upregulated (the discretized flux/proteomics activity state is higher than the discretized transcriptomics state), and about 35 are predicted to be TL-downregulated (the discretized flux/proteomics activity state is lower than the discretized transcriptomics state) (SI Table 3). The specific pathways that are predicted to be TR (high/low/moderate) and TL (up/down) regulated are listed in SI Table 6(a–c).

### Step 2: Identifying post-translational (PTL) regulated reactions

To identify the reactions that are post-translationally (PTL) regulated, we used the fluxes predicted in the previous step as a reference point. That is, reactions whose predicted flux activity markedly differed both from their transcriptomics and proteomics expression levels (that are hence not predicted to be TR or TL regulated) may be *post-translationally (PTL)-regulated*. Overall, 34, 39, 42 such reactions have at least one measured phosphorylation site in MEM, MEM-Gln and MEM+Oli, respectively. We next inferred the impact of each of the measured phosphorylation sites on enzyme activity. The phosphorylation data included 56 metabolic enzymes phosphorylated at 71 different phosphorylation sites catalyzing 164 metabolic reactions. For each of the reactions, we computed the Spearman rank correlation between the predicted flux (computed via integrating the pertaining transcriptomics and proteomics data) and the corresponding site phosphorylation levels across all growth conditions and time points measured (SI Fig. 2). 19 reactions manifested a significant p-value (<0.05) with a strong correlation (Spearman rho > |0.6|). These 19 reactions have 13 different phosphorylation sites (SI, Fig. 3).

**Figure 4:**
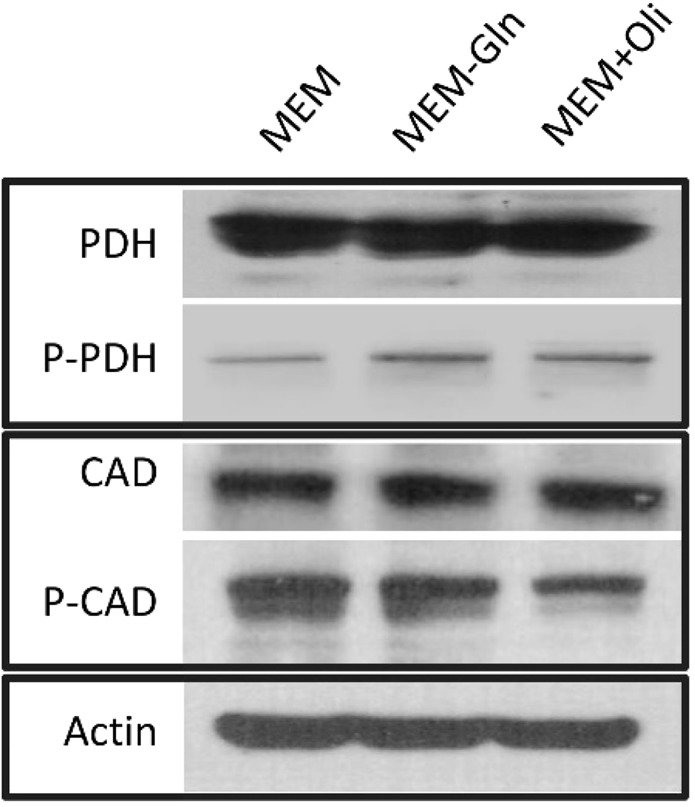
Phosphorylation of the indicated proteins (PDH and CAD) at MEM-Gln and MEM+Oli conditions were detected by western blot analysis.

The functional impact of phosphorylation is currently known from the literature for only two of these enzymes: (1) phosphorylation of S1859 in carbamoyl-phosphate synthetase 2 (CAD) enhances its *in vivo*^36^ activity, and (2) phosphorylation on S293 causes pyruvate dehydrogenase (PDHA1) enzyme inactivation^37^. Our predictions match both; for the CAD enzyme, we detected a high positive correlation (0.718) and for PDHA1 we obtained a strong negative correlation of −0.6. To test and validate these predictions in our cells further, we performed western blot experiments for both proteins (CAD and PDH together with their phosphorylated forms). We observed a marked phosphorylation of PDH in the predicted conditions for MEM-Gln and MEM+Oli compared to MEM growth condition, indicating its reduced activity under these conditions (Fig. 4). This is additionally confirmed via flux measurements through ^13^C MFA (SI, Table 1). On the other hand, we observed a decreased phosphorylation at CAD protein, indicating a decrease at its activity at MEM-Gln and MEM+Oli conditions, as predicted (Fig. 4).

### Step 3: Genome wide prediction of TR and TL regulation of breast cancer metabolism

**Figure 5:**
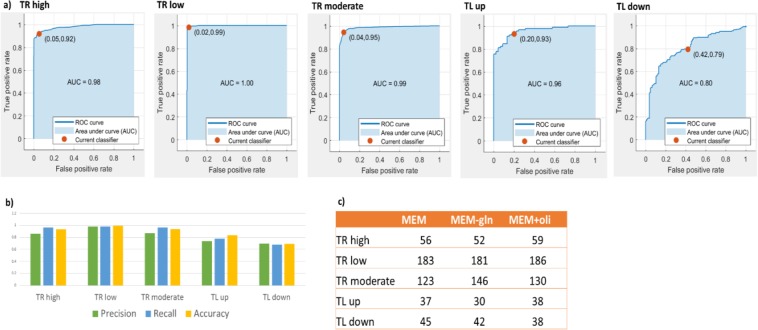
(**a**) AUC curves for each of the direct regulation SVM classifiers; (**b**) mean precision and recall values for each of the SVM classifiers; (**c**) number of reactions that have been uniquely predicted to be directly regulated by one of the classifiers.

In the previous steps, we have identified about 500 reactions that are directly regulated at one of the three regulatory levels described above (TR, TL or PTL). In these reactions, the predicted flux changes were significantly associated with molecular alterations in the pertaining enzymes. However, this leaves a large number of about 1450 reactions that were not assigned to any of these direct regulatory levels, which can be attributed to the limited scope of our measurements. In order to predict additional reactions that are likely to be directly regulated at TR or TL level, we built five Support Vector Machine (SVM) classifiers for five different direct regulation levels: TR-high, TR-low, TR-moderate, TL-up and TL-down. The goal of each classifier is to predict whether a reaction is regulated at one of these levels or not. The classifier was trained and evaluated using the reactions that have already been labeled as TR or TL regulated in the previous analysis at step (1), using a standard train and test 5-fold cross validation. The classifier input features included the gene expression, proteomics, predicted fluxes and metabolic network characteristics (reversibility information, number of participating metabolites, index of the relevant pathway, and more) of the given reactions, and the TR/TL labels already assigned in the previous steps (see methods). The accuracy of the classifier was measured by comparing the predicted labels against the known labels. The resulting classifiers achieved a high cross validation prediction accuracy (mean AUC > 0.946 for all classifiers, all values are presented in Fig. 5a; recall and precision values are presented in Fig. 5b). Applying this to predict the direct regulation of the 1450 remaining reactions, ~450 additional reactions were predicted to be regulated at exactly one of the TR/TL levels (in MEM, MEM-Gln and MEM+Oli, see Fig. 5c for their subdivision in each of the regulation groups). The predicted TR group is enriched with transcription factor binding sites (hyper-geometric p-value = 6.236e-119, see methods. Similarly, the predicted TL group has a significantly higher number of flux/proteomic states matches compared to the randomly selected sets (empiric p-value = 0.04). It is important to note that the very small numbers of predicted PTL reactions did not enable us to build reliable predictors of regulation at this level. Interestingly, adding the new set of predicted reactions which are directly regulated to those reactions which are previously identified as directly regulated by model based integration uncovers a large number of new pathways that now become enriched in directly regulated reactions (SI Table 6).

### Step 4: Studying the reactions that are indirectly regulated via stoichiometric coupling

After the predictions we performed at step 1–3, around 1000 reactions yet remained not to be predicted as directly regulated, some of which are likely to be further identified as regulated with more extensive data. However, many of these remaining unassigned reactions may still be truly *indirectly regulated (IR)* reactions where their flux may be primarily metabolically-regulated by changes in their substrate and product levels due to changes in the flux activities of other reactions in the metabolic network. That is, their flux may be *stoichiometric coupling (SC-regulated)* to the flux of other reactions in the metabolic network^38–40^.

In the framework of MCA (Metabolic Control Analysis), it has been established that network structure is an important determinant of metabolic control^41^. Accordingly, a perturbation in enzyme abundance or activity can be propagated through reactions stoichiometry coupled to the reaction catalyzed by such enzyme. To study such dependencies on a genome-scale, we used flux sampling to quantify the pairwise stoichiometric couplings between all the metabolic reactions in the human network, identifying for each reaction how tightly its flux is coupled to the flux of each of the other reactions, in each of the different conditions (see Methods).

Remarkably, we found that the ~1000 ‘unassigned’ indirect reactions have significantly higher stoichiometric couplings to the TL and PTL directly regulated reactions than among themselves across the different growth conditions (using one sided Wilcoxon test, p-values = 6.9163e-158 and 2.945e-14, respectively). These findings point out that the regulation of cellular metabolism may be governed in a hierarchical manner where the flux of many indirectly regulated reactions is determined via stoichiometric coupling to the flux of others, directly regulated reactions. Finally, we found that the group of ~1000 indirectly regulated reactions is highly enriched with bi-directional reactions (hyper-geometric p-value = 1.15e-28, 2.21e-32, 5.54e-32 for each condition, see Methods). This observation can be explained by metabolic control analysis (MCA)^42^ theory: In the framework of MCA, enzyme activities catalyzing reversible reactions, which often are in rapid equilibrium, usually have low flux control coefficients and hence are poor targets of direct regulation. Indeed, the combination of the ‘directional flexibility’ of candidate SC-regulated reactions with their enhanced coupling to other directly-regulated reactions is likely to facilitate the formation of stoichiometrically feasible flux distributions across the metabolic network, providing a way for efficiently regulating the metabolic state with minimal cellular costs in terms of transcriptomics, proteomics and phospho-proteomics regulation.

## Discussion

This study integrates transcriptomics, proteomics, phospho-proteomics and fluxomics data with metabolic modeling to provide the first chart of metabolic regulation in MCF7 breast cancer cells on genome scale. We classified the metabolic enzymes as those that are predicted to be directly regulated at three distinct levels (TR, TL, and PTL) and those that are predicted to be indirectly regulated, given the current coverage of omics data. As expected, we found that citric acid cycle is generally upregulated both on the transcription and translational level. Interestingly, while on the transcriptional level fatty acid oxidation was found to be generally down-regulated, it is up-regulated on the translational level. In addition, oxidative phosphorylation – another hallmark of cancer, was found to be up-regulated only on the translational level (not including MEM+Oli medium). These findings further highlight the pivotal role of translational regulation in cancer and the importance of obtaining higher coverage of proteomic data, whenever possible.

Remarkably, we found that the flux of the indirectly regulated reactions is coupled to the flux of directly regulated ones. We also found that the indirectly regulated reactions are enriched with bi-directional reactions. These findings might open an opportunity for further research to determine an extent by which their activity levels are set by other reactions. Taken all together, these findings suggest that the regulation of breast cancer cell metabolism is controlled in a hierarchical manner where the direct regulation of about half of the reactions suffices to orchestrate the flux regulation through the whole metabolic network via flux coupling.

Like almost any other computational, genome scale investigation, our approach has quite a few limitations. First, the data itself, is still limited and noisy, and the coverage of different layers of omics data is uneven, due to obvious technical limitations. Second, guided by the data we collected, we focused here on studying post-translational modifications mediated by phosphorylation. However, obviously, post-translational modifications occur via a variety of additional mechanisms, including, e.g., acetylation, glycosylation and allosteric regulation^43,44^. Consequently, the machine learning predictors built for predicting transcriptional regulation and post-transcriptional regulation, but not post-translational regulation. Fourthly, as we employ coarse discretization to overcome some of the noise in the data, we only identify regulatory alterations in reactions that are differentially active across the conditions of study. This limitation is partly mitigated, however, by analyzing three very distinct metabolic states. Future work should aim to address these limitations by incorporating data sets covering more conditions, measuring a wider range of omics data with higher coverage, and ideally, move to perform such measurements in patients’ tumor data. With the advent of omics technologies such data may become readily available soon and may be benefit from the conceptual and computational framework laid out in the current study.

Although we analyzed multiple layers of omics data, their coverage has been limited: while we had gene expression data for all 1372 metabolic genes, the coverage of our cutting-edge proteomics measurements provided data for only 486 metabolic enzymes and 71 of their phosphorylation sites. Flux measurements using ^13^C labeling are understandably even more limited in their scope, covering only central carbon metabolism. Aiming to make the best use of the available data and to obtain a genome-wide view of breast cancer cell metabolism, we used a modeling approach to integrate the data and infer the most likely genome-scale flux distributions. Additional work aiming to deal with the limited coverage problem was carried out via creating SVM predictors that used the known network properties together with measurements with high coverage and helped us extend the scope of the study to the utmost. With rapid advancement of high-throughput technology and accumulation of more comprehensive omics data across additional cellular conditions, the conceptual and computational framework exhibited here lays the methodological foundations for gradually obtaining a more comprehensive view of metabolic regulation in both breast cancer and other cancer types.

## Materials and Methods

### Cell culture

Breast cancer cell line, MCF7 was purchased from ATCC and cultured in MEM without phenol red (Gibco, Thermo Fisher Scientific Inc., Waltham, MA, USA) containing 10% Fetal Bovine Serum (Sigma), 10 mM d-glucose (Sigma-Aldrich), 1 mM sodium pyruvate (Biological Industries), 2 mM glutamine (Gibco), 0.1% antibiotic (penicillin 10 Units/ml-streptomycin 10 Units/ml, Gibco), 0.01 mg/ml insulin (Sigma), and 1% non-essential amino acids (Biological Industries). The cells were maintained at 37 °C with 5% CO_2_ and saturated humidity. Growth medium was replaced every 2–3 days.

Many breast cancer cells, including MCF7, display glutamine addiction habits; that is, they rely on glutamine as the main source of energy rather than glucose^45^. Besides that, they also have elevated mitochondrial activity, and considering that hypoxia is a common condition in the tumor microenvironment, the study of metabolism in the presence of strong stress condition such as hypoxia is also particularly interesting. Therefore; to study the regulation of breast cancer cells, we applied these two perturbations; glutamine deprivation and mitochondrial inhibition by oligomycin.

For the experiments, MCF7 cells were seeded and 48 h later, the medium was exchanged with an adaptation medium, MEM without phenol red (Gibco) containing 10% dialyzed Fetal Bovine Serum (Sigma) and the above-mentioned supplements. For the metabolomics experiments, after 24 h of incubation with adaptation medium, for the MEM systems, the medium was exchanged with the same medium containing 10 mM [1,2-^13^C_2_]-glucose (Sigma) or 4 mM [U-^13^C_5_]-glutamine (Sigma) with or without oligomycin (1 µM). For the MEM-Gln systems the replaced growth medium did not contain glutamine but only 10 mM [1,2-^13^C_2_]-glucose (Sigma). The cells were counted at 0 h, 8 h and 24 h after tracer introduction, and cell pellet and media were immediately frozen to use in later analysis. For the proteomic experiments heavy labeled MCF7 cells were used as an internal standard. To obtain complete labeling, cells were cultured in DMEM deprived of lysine and arginine, and supplemented with the heavy versions of these amino acids, ^13^C_6_^15^N_2_-lysine (Lys8) and ^13^C_6_^15^N_4_-arginine (Arg10). After ten cell doublings, complete labeling was achieved and validated by mass spectrometric analysis.

### Biochemical assays

Glucose, lactate, glutamine and glutamate concentrations were determined by spectrophotometry (COBAS Mira Plus, Horiba ABX) from frozen cell culture medium as previously described^46–48^. Briefly, extracellular glucose was measured by calculating the NAD(P)H concentration decrease after the conversion of total glucose by hexokinase and conversion of resulting glucose-6-phosphate into D-gluconate-6-phosphate by G6PDH using coupled enzymatic reactions (ABX Pentra Glucose HK CP, HORIBA ABX, Montpellier, France). Lactate concentration was determined by lactate dehydrogenase (LDH) reaction and measurement of NADH change. Similarly, the glutamate concentration was determined by glutamate dehydrogenase (GDH) reaction and measurement of NADH change. To measure glutamine concentration, glutamine was first converted to glutamate by glutaminase (GLS) reaction and then glutamate concentration was quantified as described above. Consumption and production rates of metabolites in the cells were analyzed by measuring the decrease or increase in concentration of the extracellular metabolites in the media at 8 h or 24 h compared to the initial concentration of the metabolite, with respect to the total cell number at each time point.

### **13**C Assisted metabolomics

Isotopologue distribution analysis of intracellular and extracellular metabolites was performed by gas chromatography coupled to mass spectrometry (GC-MS). All GC-MS analysis was carried out using an Agilent 7890 A GC equipped with HP5 capillary column connected to an Agilent 5975 C MS. GC-MS analysis of fatty acids was carried out using a GCMS-QP 2012 Shimadzu coupled with bpx70 (SGE) column. For all measurements, 1 µL of sample was injected at 250 °C, helium as the carrier gas, at a flow rate of 1 mL per minute. Each metabolite or metabolite set had different isolation, derivatization and detection procedures as explained in^49–51^. Raw mass spectra of metabolites were corrected for natural abundance of ^13^C, ^29^Si, ^30^Si, ^33^S, ^34^S to compute the fractions of ^13^C incorporated into the analyzed metabolic products from artificially labeled substrates. Data are available via Metabolights with identifier MTBLS183. (https://www.ebi.ac.uk/metabolights)

### 13C Metabolic flux analysis (13C MFA)

Our in-house developed software, Isodyn [https://github.com/seliv55/isodyn], was used to simulate the transfer of the tracers from [1,2-^13^C_2_]-glucose or [U-^13^C_5_]-glutamine medium into intracellular metabolites. Isodyn is a program written in C++ and designed to simulate the dynamics of metabolite labeling by stable isotopic tracers^18–22^. This program automatically constructs and solves a large system of ordinary differential equations which describe the evolution of isotopologue concentrations of metabolites produced in glycolysis, TCA cycle and PPP. Initially, all the metabolites except for introduced labeled substrates with known isotopologue composition in the medium are considered to be non-labeled and initial total concentrations of intracellular metabolites are calculated as a function of model parameters assuming a steady state at the initial moment. There is a function designed specifically for each type of reaction (i.e. carboxylation, decarboxylation) and these functions simulate transformation of carbon skeleton (specific transition of labeled carbon) and consumption and production rates of each isotopologue in the considered system. These transformations redistribute ^13^C isotopes in all metabolites, so that, individual rates which determine the values of the derivatives for the isotopologues are calculated for each isotopologue. To solve this system, a method of numerical integration is chosen arbitrarily (Runge-Kutta, BDF, Dassl). Isodyn simulates a real-time course of label propagation starting from the initial values of experimental conditions of incubation. As it compares the experimental and computed data for corresponding time points, reaching an isotopic steady state is not necessary.

### Western blot

Cell extracts were obtained from fresh plates 24 h after incubation with the corresponding growth medium. Then, cells were incubated for 30 min on ice with lysis buffer, scraped, sonicated and centrifuged at 15,000 g for 20 minutes at 4 °C. Supernatants were recovered and the protein content was quantified by the BCA kit (Pierce Biotechnology). Western blot analysis was carried out size-separating an equal amount of protein by electrophoresis on SDS polyacrylamide gels, and then the proteins were electroblotted onto polyvinylidene fluoride transfer membranes (PVDF) (Bio-Rad Laboratories, Hercules, CA, USA). The membranes were blocked with 5% of non-fat dry milk in PBS with 0.1% Tween, and then incubated with specific primary antibodies overnight at 4 °C. Next, membranes were treated with the appropriate secondary antibody for 1 hour at room temperature. All blots were visualized on Fujifilm X-ray (VWR International, Radnor, PA, USA) with chemiluminescence detection using Immobilon ECL Western Blotting Detection Kit Reagent (EMD Millipore, Billerica, MA, USA). The antibodies used were CAD (Santacruz Biotechnology), CAD-P (Cell Signaling), PDH (Merck Millipore) PDH-P (Cell signaling) and β-actin (MP Biomedicals). Also, anti-mouse (Dako) and, Anti-rabbit (Amersham Biosciences) secondary antibodies were used.

### Transcriptomics analysis

mRNA was extracted from cells using GeneAll Hybrid miRNA kit according to manufacturer instructions. mRNA was then processed on Atlas machine using Affymetrix Human Gene 2.1 ST Array Strip and WT expression kit. CEL files were analyzed using Affymetrix Expression Console software. The data were converted to log2 RMA values.

### Proteomics and phosphoproteomics analysis

MCF7 cells were lysed in buffer containing 4% SDS, 100 mM DTT in Tris-HCl pH 7.5. Equal protein amounts were combined with the SILAC standard and 5–10 mg proteins were digested using the FASP protocol^52^. From each sample, 10 ug were taken for proteomic analysis, and the rest was used for phospho-peptide enrichment with IMAC. Single runs were performed for each proteomic and phospho-proteomic sample.

MS analysis was performed on the EASY-nLC1000 nano-HPLC coupled to the Q-Exactive MS (Thermo Scientific). Peptides were separated on PepMap C18 columns using 200 min gradients. Raw MS files were analyzed with MaxQuant. Database search was performed with the Andromeda search engine using the Uniprot database. A decoy database was used to determine a 1% FDR cutoff on the peptide and protein levels. For phospho-proteomic analysis, the database search included p(STY) sites as variable modifications. Data are available via ProteomeXchange with identifier PXD006449 (http://www.proteomexchange.org/)

### Genome-scale metabolic modeling (GSMM)

A metabolic network consisting of *m* metabolites and n reactions can be represented by a stoichiometric matrix *S*, where the entry *S*_*ij*_ represents the stoichiometric coefficient of metabolite *i* in reaction *j*^53^. A GSMM model imposes mass balance, directionality, and flux capacity constraints on the space of possible fluxes in the metabolic network’s reactions through a set of linear equations:

$$S\cdot v=0$$

$${v}_{{\min }}\le v\le {v}_{{\max }}$$

where *v* stands for the flux vector for all of the reactions in the model (i.e. the flux distribution). The exchange of metabolites with the environment is represented as a set of exchange (transport) reactions, enabling a pre-defined set of metabolites to be either taken up or secreted from the growth media. The steady-state assumption represented in equation (1) constrains the production rate of each metabolite to be equal to its consumption rate. Enzymatic directionality and flux capacity constraints define lower and upper bounds on the fluxes and are embedded in equation (2).

In the following, flux vectors satisfying these conditions will be referred to as feasible steady-state flux distributions.

### Pathway enrichment analysis

Based on iMAT results, which was used to predict the regulation of the reactions in the metabolic model, a hypergeometric p-value was computed for each pathway in the model for being enriched with reactions that are regulated in each level. Data for reactions and their pathways were taken from BIGG database^54^. A correction for multiple hypotheses was done using false discovery rate method of 0.05.

### Using iMAT with transcriptomics and proteomics as its input

We first employed a discrete representation of significantly high or low enzyme-expression levels across tissues. Gene expression and proteomics levels were discretized to highly (1), lowly (-1), or moderately (0) expressed, for each sample. This discretization was conducted as follows: the 1/3 of the proteomics with the highest values to be considered as highly expressed, and vice versa for lowly expressed. When proteomics data was not available, transcriptomics data was used (again – top 1/3 as lowly expressed, and vice versa). One could argue that the different levels of coverage between transcriptomics and proteomics could suggest using different thresholds for determining ‘active’ and ‘inactive’ genes in the respective analysis; To keep a systematic approach, here we opted to treat both data measurements in the same, uniform, way (but other approaches may be taken in the future. Lastly, in order to avoid direct effect of the coverage differences between proteomics and transcriptomics, we determined a moderate expression level for genes whose level according to the gene expression was high (low) and according to the proteomics low (high), and left their corresponding enzymes/reactions unconstrained. In iMAT analysis, the discretized gene expression levels were incorporated into the metabolic model to predict a set of high and low activity reactions. Network integration was done by mapping the genes to the reactions according to the metabolic model (see methods), and by solving a constraint-based modeling (CBM) optimization problem to find a steady-state metabolic flux distribution. CBM models the cell as a network of metabolic reactions controlled by hundreds of genes and enables the prediction of feasible metabolic behavior under different genetic and environmental conditions, that are expressed as constraints in the network^55,56^. By using the CBM approach, we assign permissible flux ranges to all the reactions in the network, in a way that satisfies the stoichiometric and thermodynamic constraints embedded in the model and maximizes the number of reactions whose activity is consistent with their expression state. iMAT’s solution may not be unique as a space of alternative optimal solutions (in terms of its objective function) may exist. Therefore, we sampled 2,000 different flux distributions that are all consistent with the reactions’ state of activity or inactivity defined in one of iMAT’s optimal solutions. To address the potential degeneracy of the CBM solutions, we used the artificial-center-hit-and-run (ACHR) sampling approach^57^ which is an efficient sampling approach for a linearly constrained space^58^ (mean, min and max flux and flux range for each reaction is provided in SI). The mean flux distribution obtained over the 2,000 samples then serves as an approximation of the source metabolic state.

### Gene to reaction mapping

To map the gene expression to expression on the reaction level, we used the boolean gene-protein-reaction (GPR) associations available in the H. sapiens recon1 metabolic model, downloaded from the BIGG database (52). These rules indicate which genes need to be expressed using the two Boolean operators “and” and “or”. An example of such a rule is the following:

R1 = (g1 or g2) and g3 (indicating that either gene 1 or gene 2 (or both) need to be expressed in combination with gene 3 to allow reaction 1 activity.

OR rules were converted to the maximum transcription level of either of the genes, i.e. (g1 or g2) was converted to max(g1, g2)

AND rules were converted to the minimum transcription level of either of the genes, i.e. (g1 and g2) was converted to min(g1, g2).

### Bi-directional reactions

Bi-directional reactions are those that can potentially carry flux in both directions (this information is provided in the human GSMM model).

### Identifying TR/TL reactions

We compared the discretized gene expression measurements to the activity levels of the predicted fluxes; we took 1/3 of the reactions with the highest flux values to be considered as highly active, and vice versa for lowly active reactions. The rest of the reactions considered to be moderately active. If the activity level of a reaction matches the discretized value according to the gene expression, in at least 3 out of the 4 cell line replicates, the reaction is considered to be TR. For the rest of the reactions, if the activity level of a reaction matches the discretized value according to the proteomics, the reaction is considered to be TL.

### Identifying PTL reaction

Among the reactions that haven’t been classified as TR or TL in the way that mentioned above, we found the sub group of reactions that were associated with at least one phosphorylation site. Reactions whose predicted flux activity markedly differed from their transcriptomics or proteomics expression levels, and that were associated with at least one phosphorylation site in 3 of the 4 cell line replications, were predicted to be potentially post-translationally (PTL) regulated.

### Finding transcription factor enrichment

First, we found the reactions that were predicted to be TR in all condition. Then, using the reaction-gene matrix, we found the list of genes that catalyze this group of reactions. Using ENRICHR tool^29,30^, we found how many of the genes have (at least one) TFs that bind to their promoter region, from exploring Jaspar^31^, Transfar^32^, ChEA^33^ and ENCODE^34,35^ databases. Same for all model genes. These values were used in the hypergeometric calculation.

### Support vector machine (SVM) classification

We built and trained five SVMs classifiers (representing 5 “classes” of regulation, as described in main text). We applied an SVM classifier with a quadratic kernel for each classifier, with the following features:

(1–4) gene expression measurements under 4 data points

(5–8) predicted fluxes under 4 data points

(9) A binary integer indicating if the reaction is reversible.

(10) An integer value associated with a unique metabolic pathway.

(11) The total number of metabolites participating in the reaction.

(12) The total number of substrates participating in the reaction.

(13) The total number of products participating in the reaction.

For the labels, we used the classification of the reactions from the previous steps (1 if it’s regulated at that level, 0 otherwise). All SVM classifiers were trained on part of this data, and later tested on all data (mean recall and precision values presented in the text).

Cross-validation was performed by setting aside one fifth of the regulated-predicted reactions in the training set. The classifier was trained on the remaining four. The classifier’s accuracy was measured by comparing the predicted labels against the known labels.

### Computing pairwise flux correlations

For each growth condition, we found 2000 different flux distributions using flux balance analysis. Then, for each pair of reactions, we calculated the Spearman correlation between their flux values. For the coupling calculations, we used the absolute values of these correlations (as coupling between reactions can be either positive or negative).

### Multiple hypotheses correction

Throughout our paper P-values were filtered by False Discovery Rate (FDR) to correct for multiple testing^59^. More specifically, first, all the p-values were sorted in increasing order, P1, P2,.., Pn. Next, we filtered p-values ${\rm{pi}}$: ${\rm{pi}}$ > $\frac{i}{n}$ * 0.05.

## Supplementary information

Supplementary information

Supplementary Dataset 1
