# Supplementary material for: The landscape of tiered regulation of breast cancer cell metabolism
Source: Sci Rep. 2019 Nov 28;9:17760. doi: 10.1038/s41598-019-54221-y (PMC6882817; doi:10.1038/s41598-019-54221-y)
Supplement: Supplementary file 1 — Supplementary information [file 41598_2019_54221_MOESM1_ESM.docx]

**SUPPLEMENTAL INFORMATION**

**The landscape of tiered regulation of breast cancer cell metabolism**

Rotem Katzir^1*^, Ibrahim H. Polat^2, 4*^, Michal Harel^5^, Shir Katz^5^, Carles Foguet^2^, Vitaly A. Selivanov^2^, Philippe Sabatier^4^, Marta Cascante^2,3^, Tamar Geiger^5^ and Eytan Ruppin^6^

**Identifying transcriptionally (TR) and translationally regulated (TL) reactions**

We distinguished between 3 groups of TR reactions: *TR-high* – reactions whose encoding genes are overall highly expressed and are predicted to have high fluxes then, and *TR-low* and *TR-moderate* reactions, defined analogously. The overlap between the TR reactions in the MEM and MEM+Oli systems was ~81% for both TR-high and TR-low categories. Notably, pathways such as glycolysis and fatty acid elongation were enriched with TR–high regulated enzymes across growth conditions (hyper geometric p-value<0.05 (FDR corrected), SI Figure 2). On the other end, Bile Acid Biosynthesis was significantly enriched with TR-low regulated enzymes across all growth conditions.

About 75% of the reactions that were predicted to be TL-up regulated in MEM-gln and MEM-gln systems overlap, same for comparison between MEM+Oli and MEM-gln. The overlap observed between MEM-gln and MEM+Oli systems was lower (~62%). For TL-down regulation, overlap was relatively low for all comparisons (~68%, SI, Figure 2). We found several pathways that are enriched with predicted TL regulated reactions at specific conditions: For example, glutamate metabolism was TL-upregulated under all conditions. Pyruvate metabolism was predicted to be TL upregulated mainly within MEM growth condition due to excess glutamine and glucose in the medium. Riboflavin metabolism and Heme Biosynthesis pathways are TL-downregulated in both media without the Oligomycin treatment (MEM and MEM-Gln). Glycolysis consistently showed strong pathway enrichment in both TR-high and TL-up regulated reactions in all three conditions, testifying to the multi-faceted direct regulation of this central pathway.

**Genome wide prediction of TR and TL regulation of breast cancer metabolism**

We next further tested the new SVMs’ TR and TL predictions. While each of the reactions predicted to be TR according to the SVM was not identified as such in the model-based analysis, taken together as a group we still expect that the set of newly predicted TR reactions will show increased regulation compared to randomly chosen sets of metabolic reactions. To test this, we counted the number of data points where the activity level of the gene expression matched the activity level of the predicted fluxes for this subset and repeated this process 1000 times for random groups of reactions of similar size (Methods). The predicted TR group has indeed a significantly higher number of matching values compared to the random groups (empiric p-value = 0.018). Additionally, we found that the group of predicted TR reactions was enriched with transcription factor binding sites (using Jaspar and Transfac databases, hyper-geometric p-value = 6.236e-119, Methods), as would be expected if indeed they were TR-regulated. To validate the TL classifiers’ predictions, we repeated the test described above, this time finding the number of matches between the proteomics activity values and the predicted fluxes activity values, and found that the number of matches for the TL group was higher than the numbers obtained from random groups (empiric p-value = 0.04).

Interestingly, adding the new set of predicted directly regulated reactions to those previously identified as directly regulated by model based integration uncovers a large number of new pathways that now become enriched in directly regulated reactions, including the carnitine shuttle that was found to be enriched with TR-high reactions in all three growth conditions. Glycine, Serine, and Threonine Metabolism, tyrosine, glutathione and fatty acid metabolisms are among the pathways that are now found to be enriched with TR-low reactions in all three mediums, pentose phosphate pathway was found to be enriched with TL-up reactions and Tyrosine metabolism was found to be enriched with TL-down reactions in all three growth conditions (SI Figure 5 includes all pathways and p-values).

**Reactions with high flux variance are highly associated with phosphorylation sites**

In the next step, we aimed to explore and study the phosphorylation data. To this end, we first mapped each enzyme to all its associated reactions according to the GPR, ended up with 56 proteins that are phosphorylated in 71 different phosphorylation sites, which have been mapped to 164 metabolic reactions. Our assumption was that phosphorylation sites are needed more where the reactions’ bound is not tight enough and there is less stability, therefore, we hypothesized that reactions with higher flux variance will tend to be more phosphorylated. To test this, we first calculated the standard deviation for each of the reactions, using all the flux distribution vectors that we obtained in at the last step of predicting fluxes. Then, we divided the reactions into 2 groups: (1) reactions associated with enzymes that have been phosphorylated in at least one of the replicates, and (2) reactions associated only to enzymes that haven’t been phosphorylated at all. We observed a significantly higher variation of metabolic fluxes in group (1) (Wilcoxon rank sum p-value=3.0508e-129).


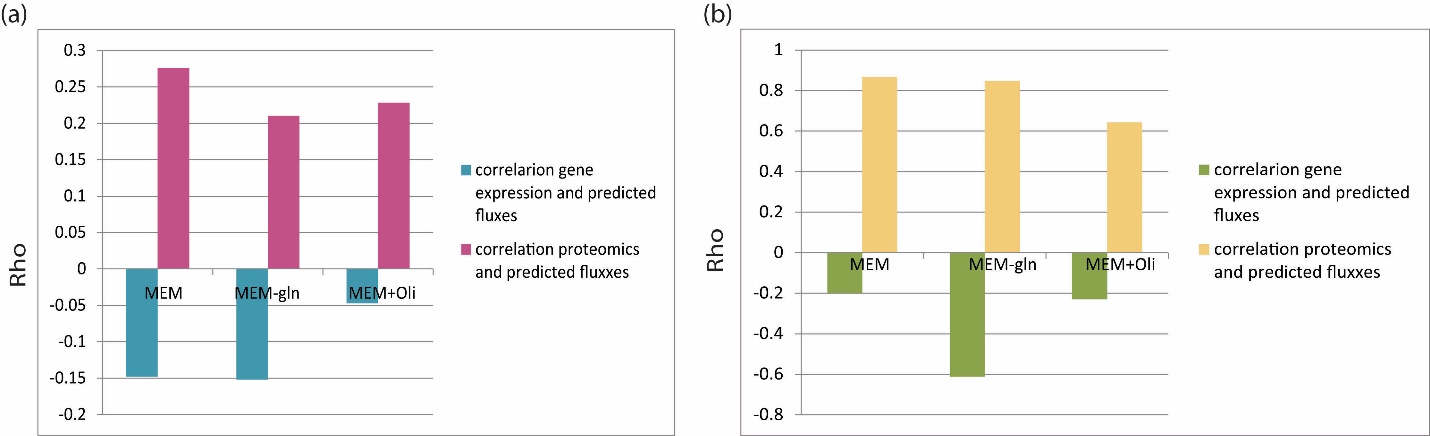


**Figure 1:** Comparison of the Spearman correlation between Gene expression and predicted fluxes, and between Proteomics data and predicted fluxes, for the subset of reactions that predicted to be (a) PTR and (b) TL.

**
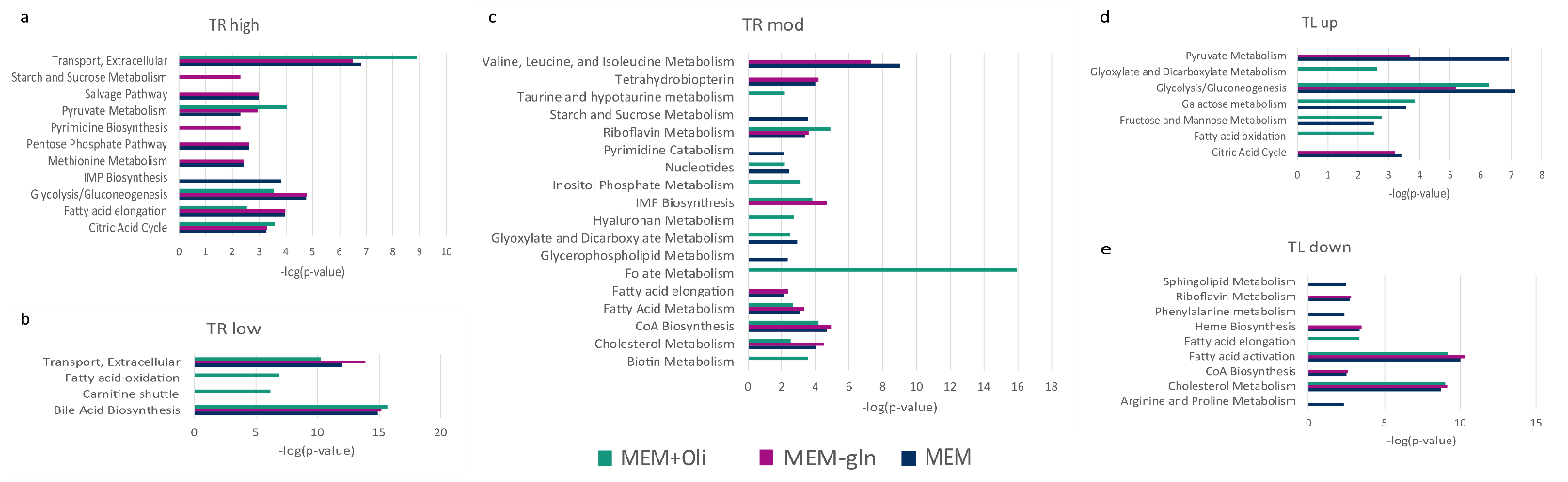
**

**Figure 2**: Pathways enriched with the reactions that are predicted as (a) TR high, (b) TR low, (c)TR moderate, (d) TL up and (e) TL down regulated (all p-values < 0.05 after FDR correction).


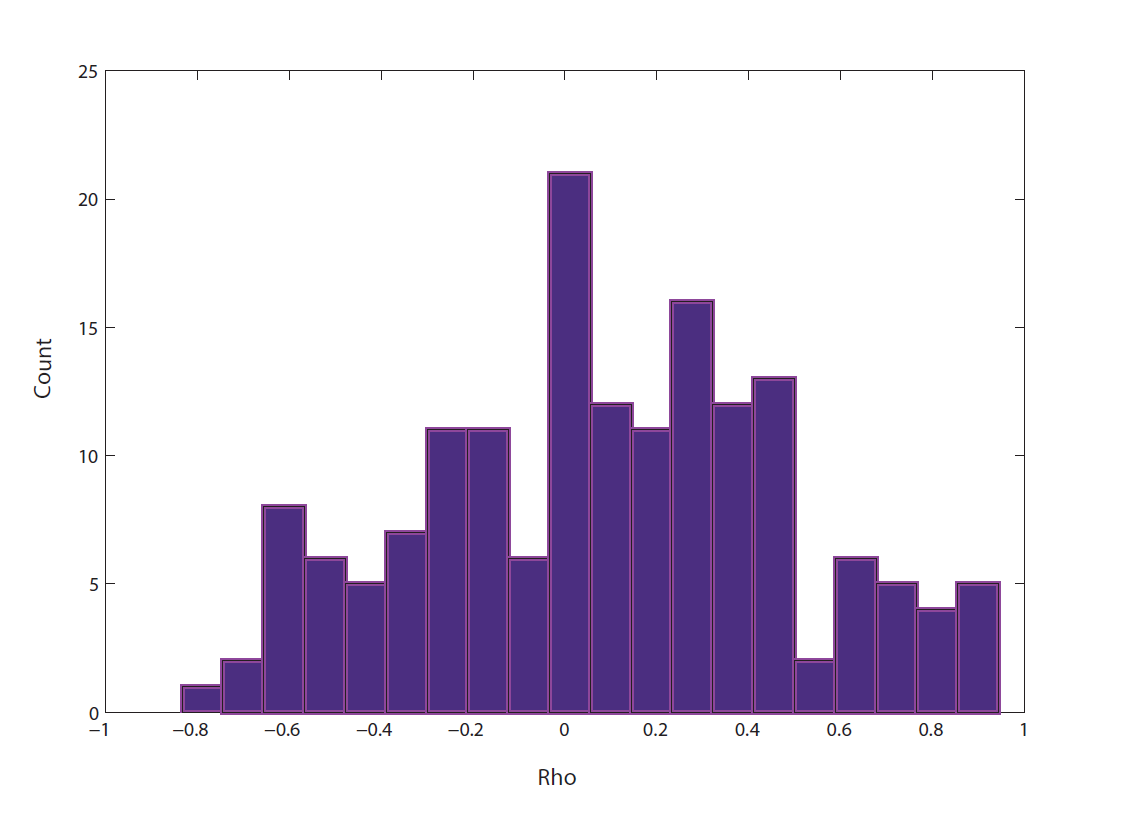


**Figure 3:** histogram of the Spearman correlation between phosphorylation and flux, for each reaction


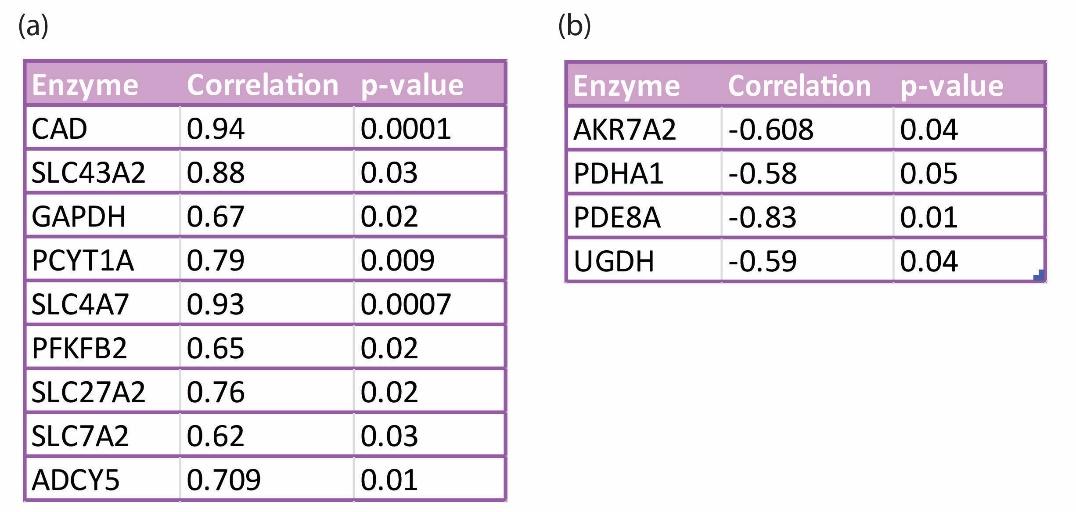


**Figure 4:** List of enzymes that having a significant (a) positive or (b) negative correlation between their phosphorylation values and their predicted fluxes values.


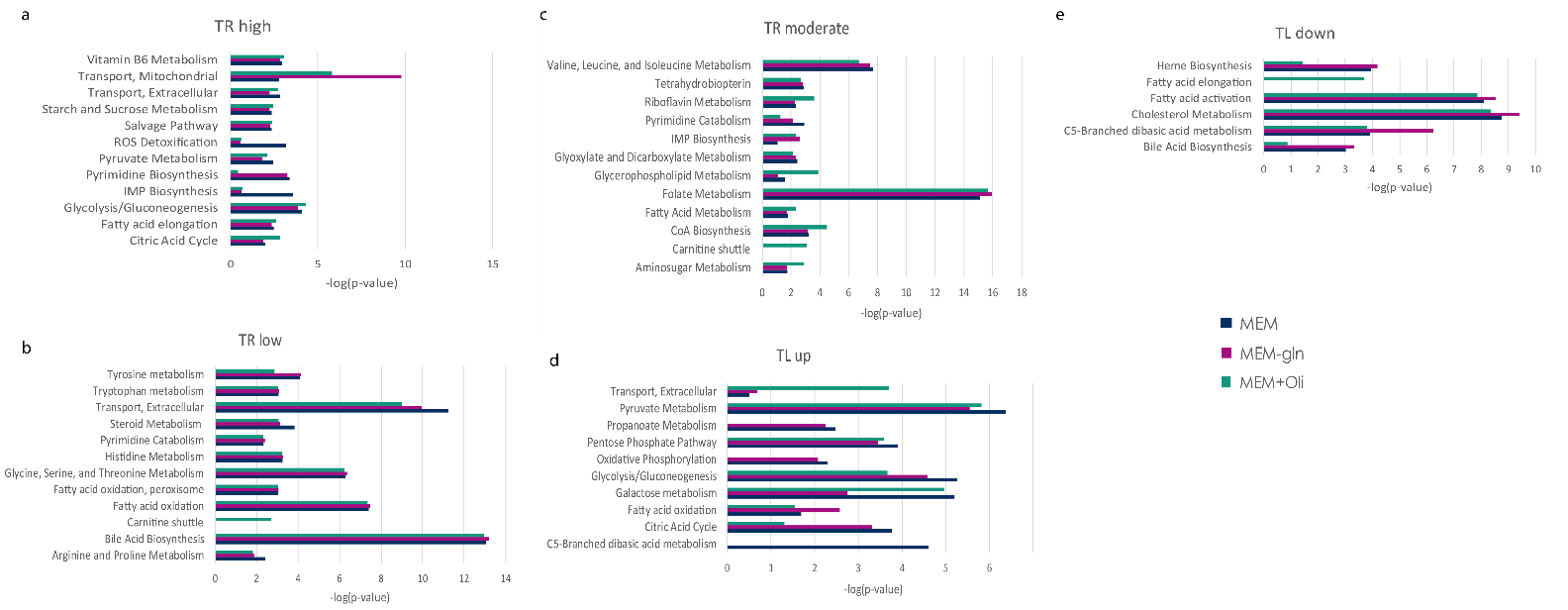


**Figure 5**: Pathways enriched with the reactions that are (a) TR high , (b) TR low, (c) TR moderate , (d) TL up and (e) TL down regulated. Regulation levels of the reactions are infered from both the GSMM model based predictions (step 1) and the predictions obtained by the SVM classifiers (step 3). Values represent the –log of the hyper-geometric p-values.


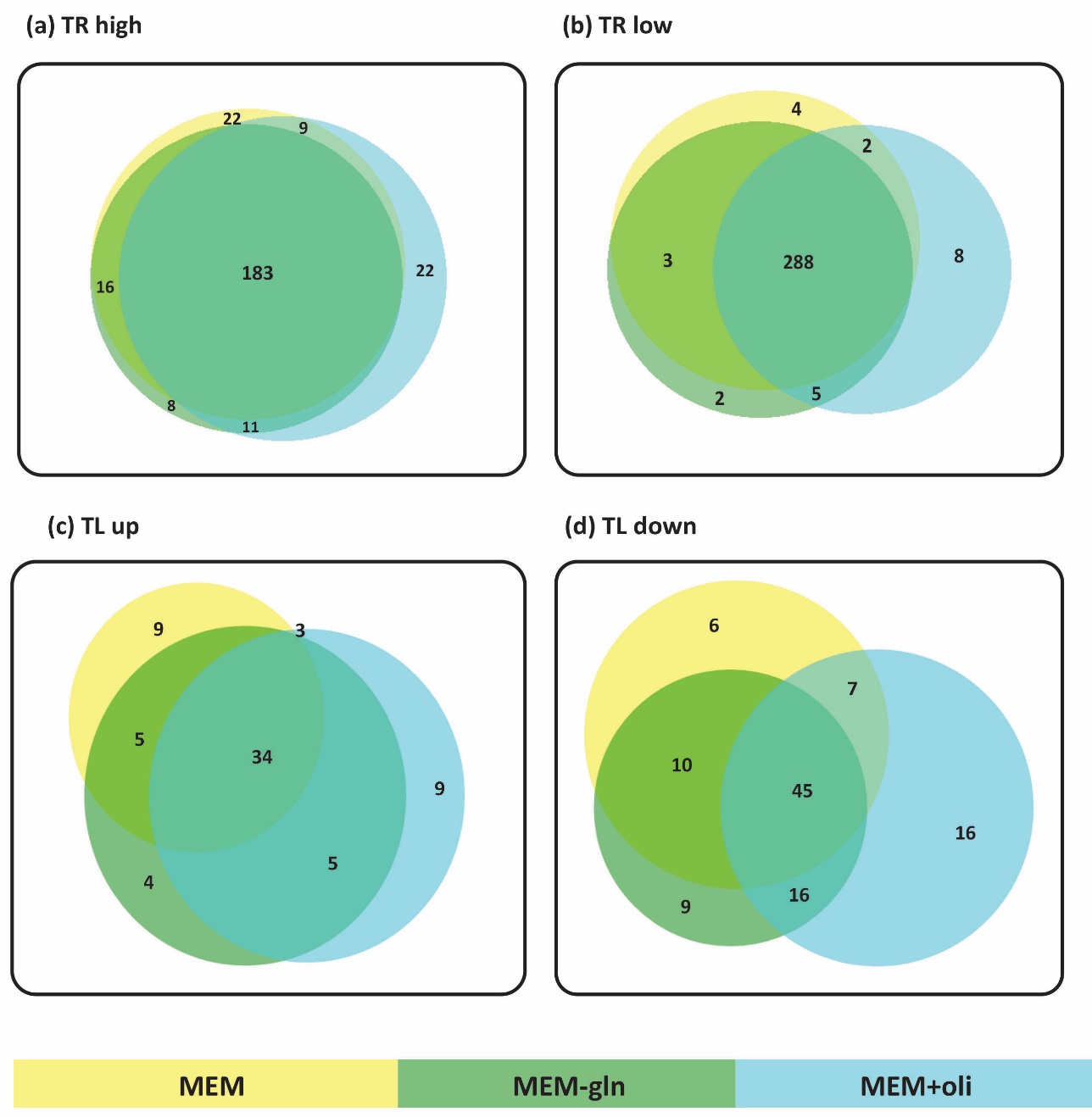


**Figure 6:** Venn diagram showing the number of (a) TR high, (b) TR low, (c) TL-up and (d) TL-down reactions, under each of the growth conditions and their overlaps.
